# Low-frequency magnetic fields potentiate plasma-modified magneto-electric nanoparticle drug loading for anticancer activity in vitro and in vivo

**DOI:** 10.1038/s41598-023-44683-6

**Published:** 2023-10-16

**Authors:** Hamed Mahdikia, Fariba Saadati, Ali Mohammad Alizadeh, Solmaz Khalighfard, Sander Bekeschus, Babak Shokri

**Affiliations:** 1 Laser and Plasma Research Institute, Shahid Beheshti University, Tehran, Iran; 2 Leibniz Institute for Plasma Science and Technology (INP), Greifswald, Germany; 3 Breast Diseases Research Center, Cancer Institute, Tehran University of Medical Sciences, Tehran, Iran; 4 Research Center on Developing Advanced Technologies, Tehran, Iran

**Keywords:** Plasma physics, Cancer, Drug delivery, Nanoparticles

## Abstract

A multiferroic nanostructure of manganese ferrite barium-titanate called magneto-electric nanoparticles (MENs) was synthesized by a co-precipitation method. FTIR, Raman spectroscopy, TEM, and X-ray diffraction confirmed the presence of spinel core and perovskite shell phases with average crystallite sizes of 70–90 nm. Magnetic, optical, and magnetoelectrical properties of MENs were investigated using VSM, UV-Vis *spectrophotometry*, DLS, and EIS spectroscopy techniques. After pre-activation by low-pressure argon (Ar) plasma, the MENs were functionalized by a highly hydrophilic acrylic acid and Oxygen (AAc+O_2_) mixture to produce COOH and C=O-rich surfaces. The loading and release of doxorubicin hydrochloride (DOX) on MENs were investigated using UV-vis and fluorescence spectrophotometry under alternating low-frequency magnetic fields. Plasma treatment enabled drug-loading control by changing the particles’ roughness as physical adsorption and creating functional groups for chemical absorption. This led to reduced metabolic activity and cell adherences associated with elevated expression of pro-apoptotic genes (BCL-2, caspase 3) in 4T1 breast cancer cells in vitro exposed to alternating current magnetic field (ACMF) compared to MENs-DOX without field exposure. ACMF-potentiated anticancer effects of MENs were validated in vivo in tumor-bearing Balb/C mice. Altogether, our results suggest potentiated drug loading of MENs showing superior anticancer activity in vitro and in vivo when combined with ACMF.

## Introduction

Over the past few decades, cancer prevalence has grown rapidly worldwide^1^. Based on the type of cancer, various cancer treatment options exist, including chemotherapy, radiation therapy, immunotherapy, and photodynamic therapy^2, 3^. There is a challenge in the chemo-drug delivery system in terms of eradicating tumor cells while sparing normal cells^4^. Targeted chemotherapy has received more attention in cancer research, using, for instance, nanomaterials as drug carriers toward tumor cells through active or passive targeting approaches^5^. In the last two decades, iron (Fe) based metal oxide nanoparticles have demonstrated substantial potential for local therapy using external magnetic fields (MF)^6^.

Multiferroic nanoparticles consist of a mixture of materials that exhibit at least two ferroic order phenomena like piezoelectricity and ferrimagnetism^7^. The coupling between piezoelectric and magnetostrictive phases allows manipulating the magnetization by an electric field (EF) or the electric polarization by an MF^8^. A new generation of devices and applications is possible due to these direct and indirect magnetoelectric (ME) effects, such as transducers^9^, nanorobots^10^, sensing phase shifting devices^11^, spintronic^12^, and magnetoelectric random access memory (MERAMs)^13^. Spinel ferrites have a conventional AB_2_O_4_ formula in which B and A are iron and one of the unstable metal cations like Co, Ni, Cu, Zn, or Mn, respectively^14^. CoFe_2_O_4_ is a hard ferromagnetic spinel with large magnetostriction, while BaTiO_3_ (BTO) is a ferroelectric with high piezoelectricity. This system is also predicted to exhibit high ME voltages as it is free of critical resource elements^8^. After functionalization, the magneto-electric nanoparticles (MENs) can be introduced with hydrophilic groups such as hydroxyl and carboxylic. Thus, they can be well-dispersed in an aqueous solution, making them a promising drug carrier substance. Undoubtedly, the most popular configuration of MENs in medical applications has a core-shell structure with a CoFe_2_O_4_-BaTiO_3_ composition. They have shown a magnetoelectric coefficient of 10 V/cmOe^15–18^. In addition, the non-zero magnetic moment allows them to control drug delivery and release mechanisms using AC and DC MFs^19^. MENs have widely been used for the externally controlled on-demand release of anti-HIV drugs experimentally^20^, particle forcing into the blood-brain barrier (BBB) for mapping and stimulation^16, 17^ and target cancer therapy^21^.

Low-pressure plasma has been shown to exert excellent potential to produce pure functional groups for biomedical applications^22^^,^^23^. Plasma polymerization can generate functional groups that can confer specific surface characteristics like hydrophilicity, hydrophobicity, cytocompatibility, and resistance to bacterial growth. These properties can be tailored to meet diverse clinical requirements^23^. Acrylic acid or carboxylic acid (CH_2_=CHCOOH) polymerization can produce carboxylate-rich (-COOH) functional groups^24^. Recently there has been significant interest in this type of surface modification for nanoparticles used in biomedical fields. This is mainly due to the absence of harmful chemicals and the safety of the process for biological organs^25^. Several studies report the utilization of ammonia, nitrogen, and nitrogen/hydrogen mixture plasmas for amino functionalization of carbon-based nanoparticles^25, 26^. Viswan et al.^25^ prepared graphite-encapsulated iron oxide nanoparticles with nitrogen-containing functional groups by applying a mixture of Ar and NH_3_ plasma performed by an inductively coupled radio frequency plasma (ICP) to immobilize Escherichia coli (E. coli). Additionally, they measured the number of amino functional groups by a chemical derivatization method^25^. Furthermore, plasma-enhanced chemical vapor deposition (PECVD) with an ICP structure was used for anti-cancer coated diglyme precursor polymerization as a drug delivery system (DDS) by optimizing plasma parameters to control the release of carboplatin^27^. Consequently, plasma exposure has been suggested to increase the free surface energy of polymers. This is done by creating derivatives of carboxyl, carbonyl, ether, amine, and hydroxyl functional groups based on the precursors^23^. The free amine groups on the DOX molecule structure are highly potentiated to bond covalently to the carboxylic and carbonyl groups generated on the MEN’s surfaces^28, 29^.

The present study aimed to develop DOX-loaded perovskite-coated magnetic nanoparticles (DOX-MENs) using plasma polymerization of acrylic acid to produce a carboxyl-rich surface nanoparticles (NPs) as a drug delivery system. In this case, plasma treatment improved drug uptake by changing the surface roughness (physical adsorption) and creating functional groups (chemical absorption). Low-frequency alternating MF exposure enhanced local and controlled release of DOX's and promoted the nano-electroporation effect on the cancer cells' surface due to their intrinsic negative potential distinguishing them from normal cells. The DOX-loaded MEN’s efficacy and safety on cancer cells were investigated in vitro and in vivo.

## Results

### Physical characteristics of MENs

**Figure 1:**
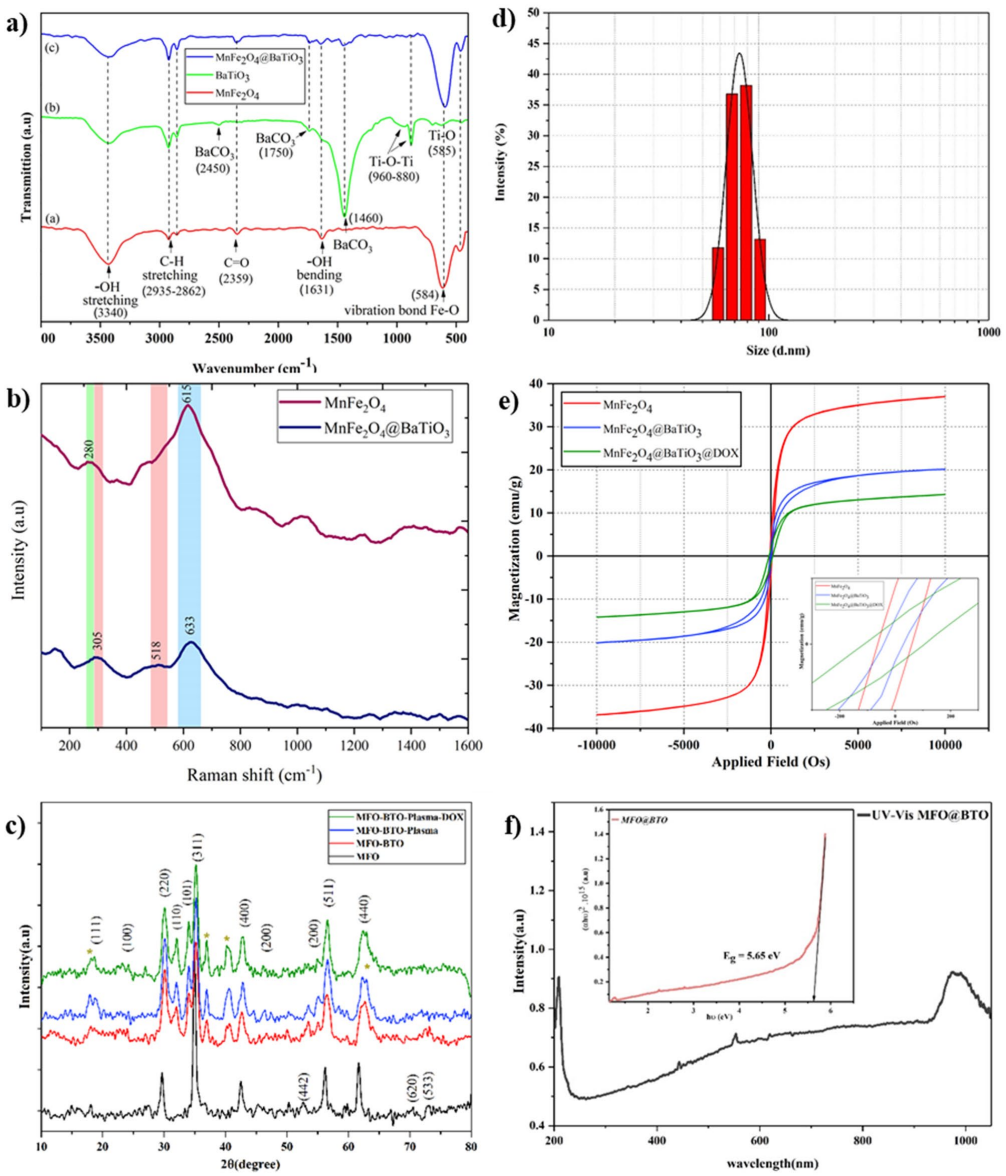
Physical characteristics of MENs. (**a**) FT-IR spectra of the MFO core, BTO shell, and MFO-BTO confirm the formation of core-shell nanoparticles; (**b**) Raman shifts of MFO and MFO-BTO confirm FTIR results; (**c**) XRD crystallography patterns of MFO, MFO-BTO, and MFO-BTO treated by plasma and loaded with DOX; (**d**) MFO-BTO particle size distribution obtained by DLS analysis; (**e**) superparamagnetic behavior and saturation magnetization of MFO, MFO-BTO, and DOX-loaded MFO-BTO; (**f**) band gap energy of MFO-BTO dissolved in ethanol obtained by UV-vis spectroscopy.

Superparamagnetic manganese ferrite nanoparticles were synthesized following a co-precipitation method and characterized using different analytical methods. FT-IR spectra of MnFe_2_O_4_ (MFO), BaTiO_3,_ and MnFe_2_O_4_@BaTiO_3_ illustrated in Fig. 1a. FT-IR spectrum of ferrite-manganese reveals a wide shoulder peak around 3420 cm^-1^ and a 1630 cm^-1^ peak, which was assigned to vibrational hydroxyl (-OH) groups. A peak at 584 cm^-1^ is attributed to the Fe-O vibration bond (Fig. 1a)^30^. Their high hydrophilic -OH group density facilitates the high aqueous dispersibility and stability of magnetic ferrite-manganese nanoparticles. This property enables them to be further utilized in functionalizing BaTiO_3_ precursors. According to the (b) spectrum in Fig. 1a, two obvious peaks at 2950 cm^-1^ and 2860 cm^−1^ were observed, which were assigned to C-H stretching bands. The vibrational peak assigned to 585 cm^−1^, 1460 cm^−1^, 1750 cm^−1^, and 2450 cm^−1^ are features of BaTiO_3._ The peaks at 880 cm^−1^ and 960 cm^−1^ are due to Ti-O-Ti bending vibration along the polar axis, and the peak at 585 cm^−1^ is due to stretching vibrations. All the mentioned peaks are detectable in the core-shell MnFe_2_O_4_@BaTiO_3,_ as shown in Fig. 1a graph (c), while the peak at 585 cm^-1^ is overlapped with a strong Fe-O band.

The FT-IR results were confirmed by Raman spectroscopy which also demonstrated the intermixing of phases in the initial cores. According to Figure 1(b), which represents the Raman spectra for MnFe_2_O_4_ core and MnFe_2_O_4_@BaTiO_3_ core-shell nanoparticles, there is a wide Raman shift at a frequency around 600-630 cm^-1^ related to the A_1g_ bond obvious in both spectra assigned to MnFe_2_O_4_ and confirmed by other reports^14^. This peak showed a small red shift in the Raman spectrum when the piezoelectric shell material covered the manganese ferrites. Another detectable Raman shift assigned to MnFe_2_O_4_ appears at 280 cm^−1^^14^. The sharp bond close to 305 cm^−1^ associated with B1 and E symmetries of longitudinal optical (LO) and transverse optical (TO) phonon modes [B1, E (LO, TO)] and the high-frequency band near 715 cm^−1^ [A1, E(LO)] (overlapped with the bond related to MnFe_2_O_4_ at 633 cm^−1^) is characteristic of the ferroelectric phase with tetragonal symmetry of BaTiO_3_. The broad and intense peaks at 270 cm^−1^ (overlapped with the bond related to MnFe_2_O_4_ at 280 cm^−1^) and 304 cm^−1^ are due to vibration in the TiO_6_ group^31^. The dominant band of BaTiO_3_ assigned at 518 cm^−1^ [A1, E(TO)] is due to vibration that occurs by the displacement of oxygen atoms^32^.

The successful synthesis of MnFe_2_O_4_ spinel structures and their crystallinity are evident from the XRD pattern presented in Fig. 1c. The peaks appeared at 2θ =16.02°, 30.19°, 35.54°, 43.21°, 53.60°, 57.06^o,^ and 62.66° which represent the Bragg reflections from the (111) (220), (311), (400), (422), (511), and (440) planes respectively determine the cubic nature of nanoparticles' core, and all the peaks match well with the standard XRD pattern of MnFe_2_O_4_ nanoparticles (JCPDS card No. 10-0319). The most intense peak (110) is found at 2θ = 32.17. The peaks at 2θ = 22.21° (100), 32.17° (110), 38.98° (111), 45.59° (200), 70.32° (300), and 74.78° (310) confirm the formation of a perovskite barium titanate shell^31^. According to Debye-Scherrer’s formula ($\mathrm{D}=\frac{\mathrm{k\lambda }}{\mathrm{\beta cos\theta }}$), it was found that the average crystallite size of produced MnFe_2_O_4_ and MnFe_2_O_4_@ BaTiO_3_ nanoparticles was about 50 nm and 85 nm, respectively. Where λ is the wavelength of Cu-Kα radiation (λ = 1.54178 Å), and θ and β represent the Bragg’s angle and the full width at half maximum (FWHM) of the considered peak. The main diffraction peaks of each sample were employed for calculation.

**Figure 2:**
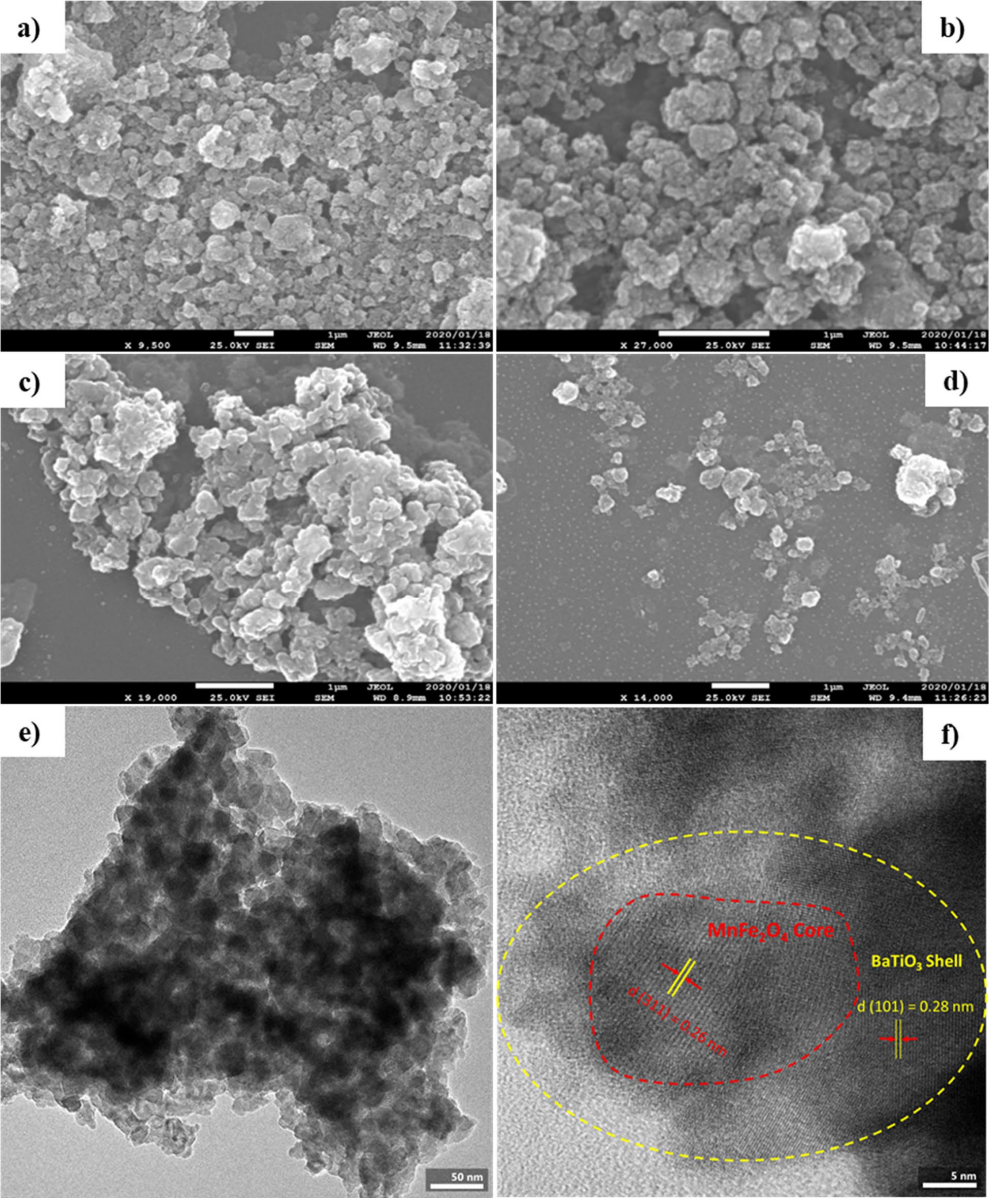
SEM and TEM images of synthesized MENs. The morphology and shape of (**a**) MFO, (**b**) MFO-BTO, (**c**) MFO-BTO plasma-treated, and (**d**) MFO-BTO-DOX. TEM images of MFO-BTO with (**e**) 50 nm and (**f**) 5 nm scale bars.

The size and morphology of MENs were determined by scanning electron microscopy (SEM) and transmission electron microscopy (TEM). Figure 2 demonstrates the FE-SEM images of MnFe_2_O_4_, MnFe_2_O_4_@BaTiO_3,_ and MnFe_2_O_4_@BaTiO_3_ functionalized by plasma and loaded with DOX. The SEM image indicates that MnFe_2_O_4_@BaTiO_3_ heterogeneous nanoparticles have a mean diameter of about 80-110 nm. The SEM and TEM images at a 1 μm scale reveals polyhedral agglomerated formations for spinel and a nearly spherical shape for the barium titanate-coated core-shell nanoparticles (Fig. 2b and e). The hydrodynamic diameter of MnFe_2_O_4_@ BaTiO_3_ was also measured by dispersing them in distilled water and ethanol solution using DLS analysis. The corresponding data were plotted in Fig. 1d. The average hydrodynamic diameter of the particles was found to be 80 nm with a size distribution in the range of 60 to 110 nm. This confirms the SEM results. The TEM image also illustrates a core-shell structure in the same dimension, as depicted in Fig. 2f. The MnFe_2_O_4_ core and BaTiO_3_ shell are characterized by their respective plane distances (d) and orientations. The plane distance of MnFe_2_O_4_, correlated with d(311), is calculated to be approximately 0.26 nm, while d(101) of BaTiO_3_ is estimated to be 0.28 nm, as shown in Fig. 2f.

The optical absorbance spectra of MENs dissolved in ethanol in the range of 200–1100 nm are illustrated in Fig. 1f. This data was used to analyze the optical bandgap energy (E_g_) according to the following formula:

$$\mathrm{\alpha h\nu }={\mathrm{c}(\mathrm{h\nu }-{\mathrm{E}}_{\mathrm{g}})}^{\mathrm{n}}$$

where c is a constant, ν is the transition frequency, and “n” is the exponent representing the nature of the band transition (n = 1/2 and n = 3/2 corresponding to indirect allowed and forbidden transitions, respectively). The optical band gap of MENs has been calculated from the intercept of the energy axis obtained by extrapolating the plot on the energy axis, as shown in Fig. 1e, and found to be around 5.65 eV.

Figure 1e shows the results of the VSM analysis. These results indicate that the saturation magnetization (Ms) of MnFe_2_O_4_ spinel is 38 emu·g^−1^. The room temperature magnetization curves of the spinel core exhibit very low hysteresis, demonstrating its superparamagnetic behavior. The Ms decreased to 20 emu·g^−1^ and 15 emu·g^−1^ by adding a piezoelectric shell and loading the drug after plasma functionalization, respectively. The inset of Fig. 1e presents a magnified view of the internal region of the hysteresis loops for all samples.

**Figure 3:**
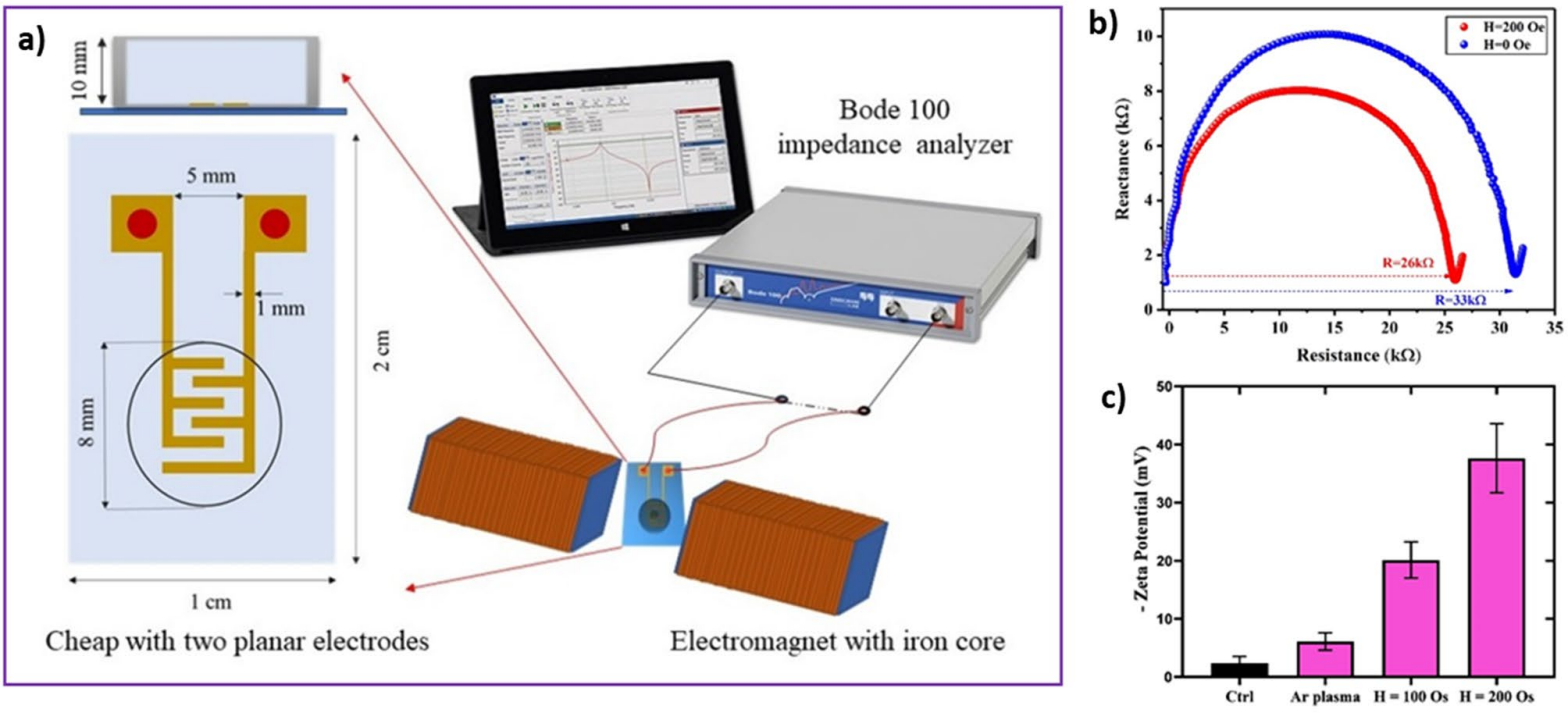
Magnetoelectric properties of MENs. (**a**) home-made chip and experimental setup of electrical impedance spectroscopy employed for MEN's impedance measurement under MF exposure; (**b**) cole-cole diagram of MENs dissolved in ethanol under MF exposure of H=0 and H=200 Oe and 100 Hz reveals a reduction in resistance of solution containing MENs; (**c**) zeta-potential of MENs determined by DLS reveals the effect of Ar plasma pre-activation (due to charge deposition on MEN's surface) and MF treatment at 100 Oe and 200 Oe.

Magnetoelectric properties of MENs can be obtained using electrical measurements to reveal their value differences with and without exposing them to ACMF. Electrical characterization of the samples is carried out using a zeta potentiometer and electrical impedance spectroscopy (EIS) methods. To illustrate the magnetoelectric effect induced by low-frequency ACMF, the zeta potential of 100 Oe and 200 Oe exposed MENs dispersed in ethanol was compared with the zeta potential of non-irradiated pre-activated MENs (Fig. 3c). The zeta potential of non-treated MENs is obtained around − 2.5 mV, while its value decreases to − 20 mV and − 37 mV by applying 100 and 200 Oe ACMF treatment, respectively. Furthermore, EIS is used to investigate the electrical behavior of MENs under MF exposure. Figure 3b shows that a well-shaped semi-circle was observed over a wide frequency range of 1 kHz to 100 MHz for MENs with and without MF exposure due to the charge transfer process at the electrode–solution interface. Figure 3b exhibits two depressed semicircles corresponding to different high- and low-frequency relaxation processes. It can be seen that the smaller charge transfer resistance of H=0 in comparison with H>0 indicates the presence of conductive ionic liquids due to the ME effect of MENs that could greatly enhance the conductivity of the electrode. The real part of the MENs’ resistivity dissolved in ethanol decreases from 33 kΩ to 26 kΩ by applying a 200 Oe ACMF.

### Plasma surface modification of MENs

**Figure 4:**
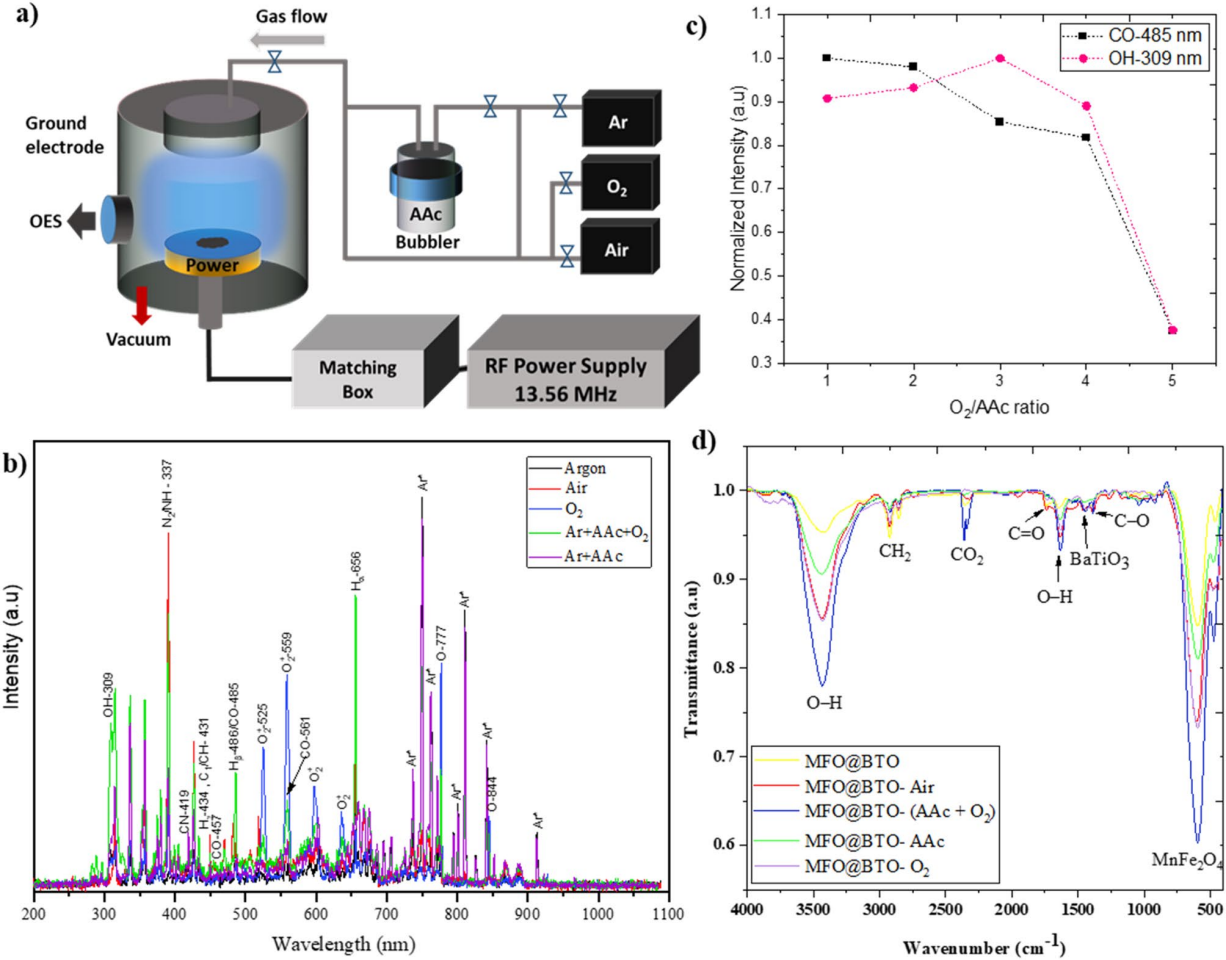
Gas-phase plasma diagnostic for MENs’ surface functionalization. (**a**) schematic illustration of low-pressure PECVD setup with CCP structure: nanoparticles were placed on a substrate on the powered electrode and exposed by Ar plasma (45 s) in CW mode and AAc+O_2_ as the precursor for (5 min) in pulsed mode; (**b**) optical emission spectra of plasma operated with Ar, air, O_2_, Ar+AAc, and Ar+AAc+O_2_ as precursor used in this study; (**c**) normalized line intensities of CO (485 nm) and OH (309 nm) of Ar+O_2_/AAc (ratio from 5:1 to 1:1) plasma; (**d**) FTIR spectrum of MENs treated with the same plasmas to investigate C=O, O–H, and C=O functional groups.

**Figure 5:**
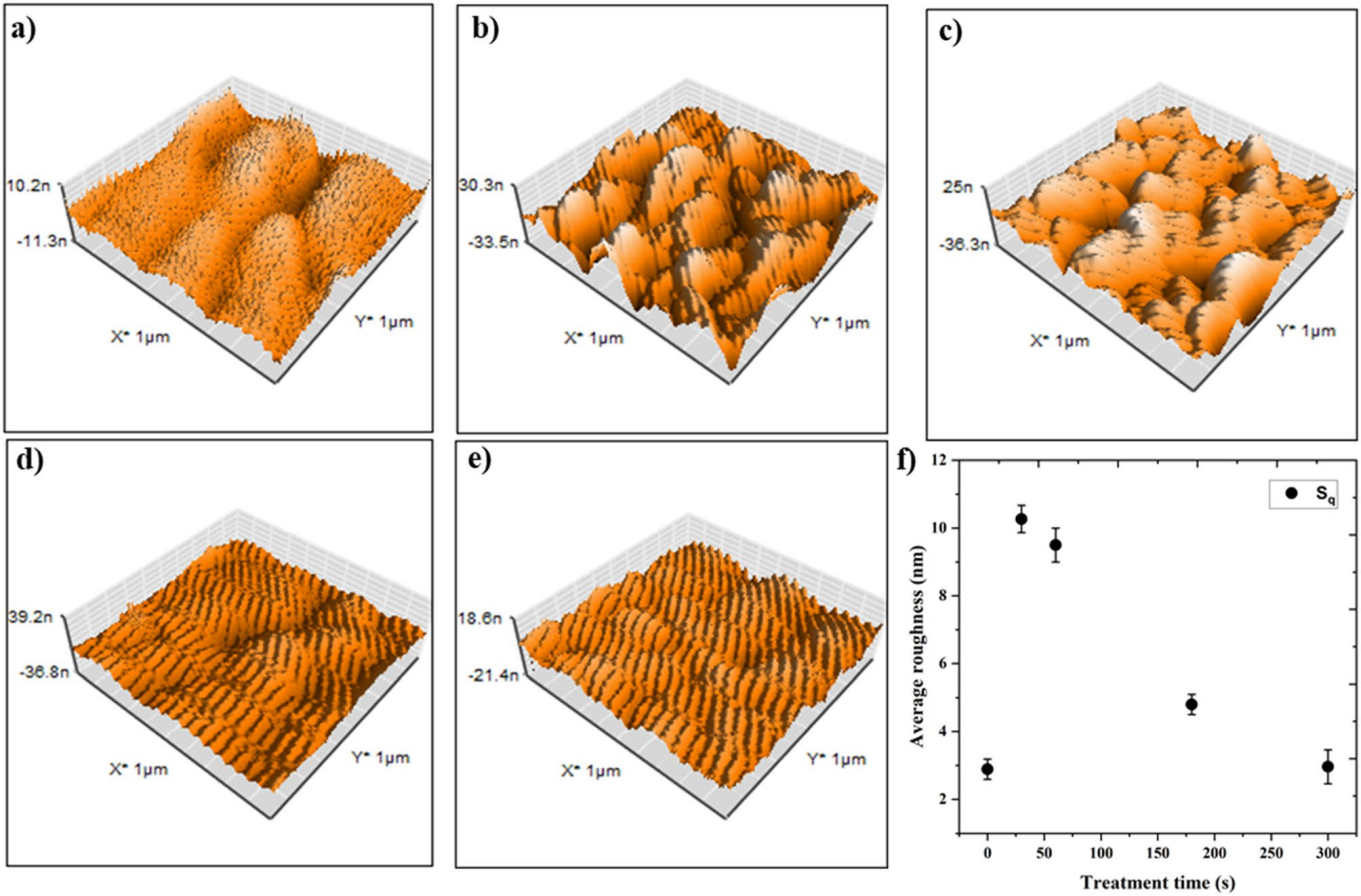
Evaluation of Ar pretreatment on BaTiO_3_ surface roughness. AFM images of BaTiO_3_ coated on quartz glass and treated with Ar plasma for (**a**) 0s, (**b**) 30s, (**c**) 60s, (**d**) 180s, and (**e**) 300s, and (**f**) the corresponding average surface roughness.

A schematic diagram of the low-pressure plasma system used in this study is illustrated in Fig. 4a. Using the same experimental conditions as for nanomaterials, BaTiO_3_ coated on glass was exposed to plasma and then analyzed using AFM to determine plasma-induced topography and surface roughness. In Fig. 5a–e, the topography results for exposure times of 0 s, 30 s, 60 s, 180 s, and 300 s are illustrated, and the root mean square roughness S_q_ is plotted in Fig. 5f. According to the results, only 30–60 s Ar pre-activation leads to significant roughness (S_q_ = 10 nm) compared to untreated BaTiO_3_ (S_q_ = 3 nm). This makes plasma exposure an ideal environment to create both physical adsorption and chemical absorption (generating functional groups) simultaneously in a liquid-free method. This means that plasma treatment of MENs shows hydrophilic behavior due to roughness, which enhances drug absorption. However, increasing exposure time decreases surface roughness. This can be explained by a long-time ion (Ar^+^) bombardment that removes a few substrate layers. In light of this result, a pre-activation of 45 s was chosen for all experiments before the main plasma polymerization.

Figure 4b shows that different gas composition discharges used in this work demonstrate distinct emission spectra. Ar, air, oxygen, AAc bubbled by Ar, and Ar +AAc mixed with oxygen are precursors to functionalize the MENs’ surface. Optical emission spectra provide insights into the plasma gas phase composition, which includes excited species generated during the treatment. As shown in Fig. 4b, besides Ar lines (mostly intensive lines at the wavelength region of 695–850 nm), other dominant species-related lines are hydroxyl band OH- 308 nm, the second positive system (C^3^Π_u_–B^3^Π_g_) of N_2_ (337 nm), CN (419 nm), H (431 nm, 486 nm, and 656 nm), CO (457 nm and 561 nm), O (777 nm and 844 nm), and some other ionized oxygen species O_2_^+^ at 525 nm and 559 nm. Mixing acrylic acid with trace gases (i.e., air) leads to emission peaks of CO at 457 nm, CN at 359 nm, and NH at 337 nm. The presence of these peaks suggests that the monomers were fragmented during plasma polymerization^33^ and reacted with impurities. Hydrogen-containing lines like the OH radical and atomic hydrogen series are more dominant in AAc-containing spectra. Meanwhile, three newly discovered lines located at 431 nm, 486 nm, and 514 nm were attributed to CH and C_2_ species. Moreover, the FTIR spectrum of the plasma-treated MENs with acrylic acid shows strong absorbance in the OH region (3600–3000 cm^-1^), with a broad band centered at 3300 cm^-1^. The absorption band of acrylic acid monomer is not present in that region. Plasma polymerization processes induce a completely new chemical structure, with additional vibration frequencies, through fragmentation and rearrangement phenomena. In the 2750–3000 cm^−1^ region, signatures of CH_x_ groups are found in all presented spectra. The spectrum of plasma polymerized AAc exhibits a dominant absorption band at 1720 cm^−1,^ which can be assigned to the stretching vibration of the C=O group, while a smaller peak at 2945 cm^−1^ is attributed to stretching vibrations of the CH bond. Therefore, our approach resulted in carboxylic functional group retention at a surface, especially in AAc+O_2_ plasma. The acrylic acid monomer absorption was not apparent due to alkene absorption bands at 1640 cm^-1^ (C=C stretch), which proved that the opening of the C=C bonds occurred during the plasma polymerization process^34^. Therefore, to improve functionality and avoid unwanted byproducts, the ratio of O_2_/AAc varied from 1 to 5, and the peak intensity of OH (309 nm) and CO (485 nm) normalized to their maximum value is plotted in Fig. 4c. As a result, 5 min treatment of a mixture of $\mathrm{Ar}+ \frac{{\mathrm{O}}_{2}}{\mathrm{AAc}}(2:1)$ after a 45s pretreatment with Ar plasma was selected as the main precursor for DOX-loaded MENs for further investigations. The short Ar plasma exposure creates maximum roughness (obtained from AFM results) suitable for physical adsorption.

### Drug loading and release

**Figure 6:**
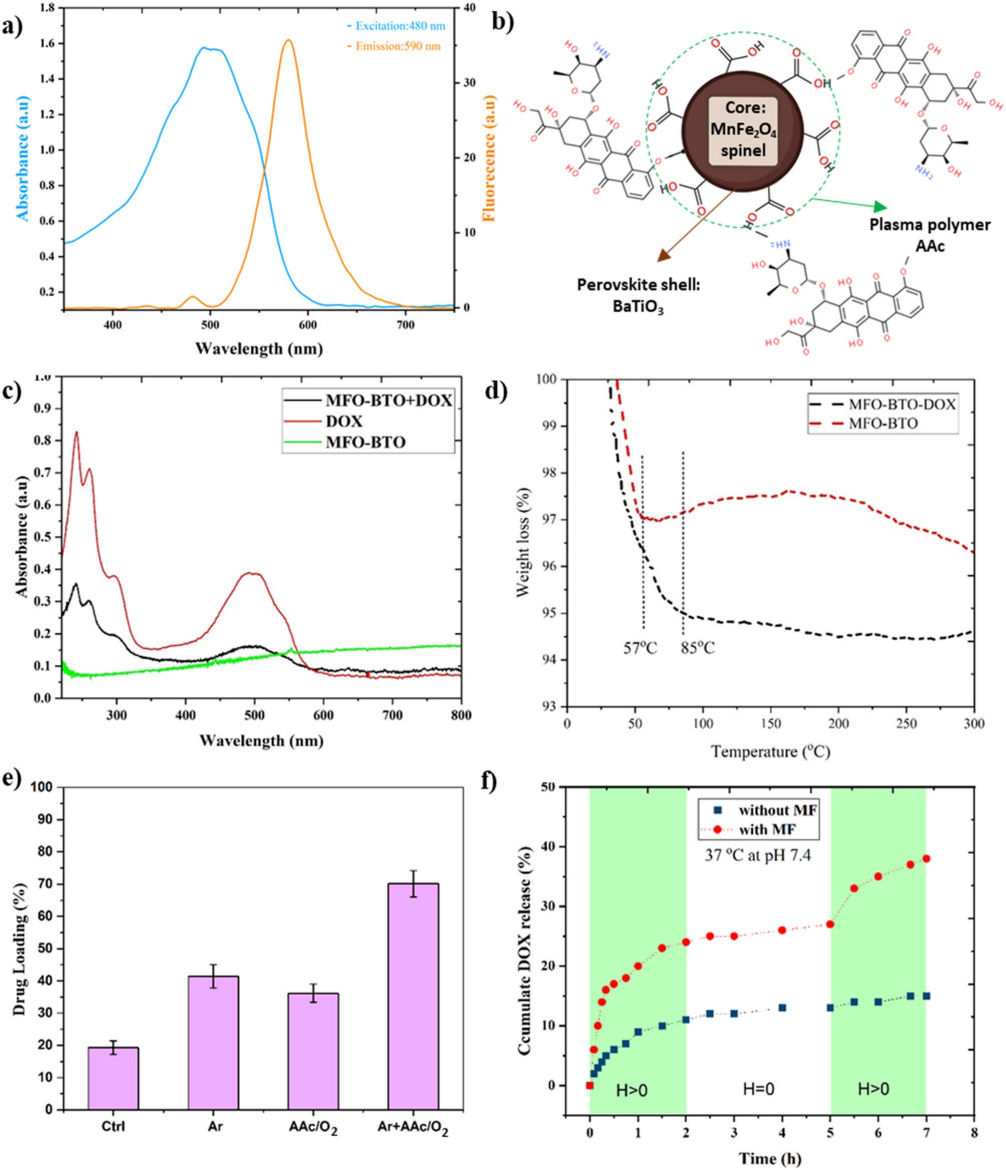
Loading and release of MENs’ DOX. (**a**) absorbance and fluorescence spectra of 2 mg/mL DOX; (**b**) schematic representation of the DOX loaded MENs with spinel core and perovskite shell, AAc plasma polymerization leads to OH, COOH, and C=O functional groups suitable to conjugate with DOX’ free bonds to improve drug uptake; (**c**) UV-vis absorbance spectra of DOX, MENs, and DOX loaded MENs confirm the drug uptake; (**d**) TGA analysis of MFO-BTO and MFO-BTO-DOX reveal an extra weight reduction due to the uptaked DOX; (**e**) Ar plasma pre-treatment, AAc polymerization, and their combined effect on the DOX loading capacity; (**f**) MF exposure effect on DOX release kinetic profiles in MENs.

Absorbance and fluorescent spectra of doxorubicin (2 mg/mL) are shown in Fig. 6a. The spectra at different concentrations were analyzed to identify the optical properties of DOX, which exhibit a wide excitation absorbance in the visible region (350–600 nm) with a max peak of 480 nm and a fluorescent emission frequency of 590 nm. To compare the drug loading capacity of different treatments on MENs, 2 mg of DOX-loaded MENs was exposed to (Ar, O_2_ + AAc, and $\mathrm{Ar}+ \frac{{\mathrm{O}}_{2}}{\mathrm{AAc}}(2:1)$) plasmas according to Table 1. A schematic illustration of the final MFO-BTO-DOX nanoformulation with spinel core and perovskite shell is shown in Fig. 6b. The plasma polymerization of AAc produces OH, COOH, and C=O functional groups suitable to conjugate with DOX-free bonds, improving the uptake of drugs. As shown in Fig. 6e, the MENs without activation can only load 18% of the free drug molecules. This is probably due to the intrinsic OH formed on the surface of the MENs during synthesis (Fig. 1a). Pretreatment with Ar for 45 s increases the MEN's loading capacity to 42% due to surface roughness changes and physical adsorption. Meanwhile, MENs treated with acrylic acid and O_2_ mixture without Ar pretreatment exhibited 37% DOX loading. Finally, pre-activation with Ar plasma followed by AAc+O_2_ deposition for 5 minutes improved drug loading capacity by 71%. DOX loading on MENs was confirmed by spectrophotometry and TGA analysis. The absorbance spectra of DOXs (at initial concentration), MENs, and DOX-loaded MENs dissolved in PBS are shown in Fig. 6c, indicating successful drug loading by plasma-treated MENs. Furthermore, TGA analyses were conducted with and without DOX added to synthesized nanoparticles. Figure 6d compares their mass loss by increasing the temperature up to 300 °C. There is a rapid reduction in weight in both samples at the first 50 °C, which is related to impurities such as moisture. However, the MFO-BTO sample's weight reduction remained constant at 97% (at 57 °C), indicating that no DOX or other components were present. MFO-BTO-DOX continued to lose weight by 95% (normalized to their initial weight) up to 85 °C. This is evident in the extra weight caused by drugs with a lower evaporation temperature. Together these results confirm that DOX was successfully loaded onto the MENs.

**Table 1:** Operating conditions for plasma functionalization.

Gas	Gas flow rate	Treatment time	Pressure	Power	Pulse mode
Ar (pre-activation)	20 sccm	45 sec	50 mTorr	75 W	CW
Air	10 sccm	5 min	60 mTorr	100 W	CW
O_2_	25 sccm	5 min	58–60 mTorr	100 W	CW
Ar (carrier) + AAc	10 sccm	5 min	57–68 mTorr	100 W	D.s (50%)
$Ar+ \frac{{O}_{2}}{AAc}(1:1-1:5)$	20 - 60 sccm	5 min	75 mTorr	100 W	D.s (50%)

*CW* continuous wave, *D.S* duty cycle.

Figure 6f shows the release profile of the MFO-BTO-DOX with and without ACMF exposure for 7 h. The ACMF (100 Oe and 100 Hz) was exposed to one of the groups for 2 × 2 h with 3 h intervals while the second group remained unexposed. Initial burst release of DOX into a release medium (PBS containing 10% ethanol (v/v), pH 7.4, 37 °C) was observed for both samples. This first rapid increase of drug released from the MENs is mostly due to the physically (non-covalent bands) adsorbed molecules on the surface, while the slower release (especially for the second group without MF) in the plateau after 2 h is more related to the chemically grafted DOX on the surface which requires more retention time under the shaking process and a normal temperature and pH. In contrast, low-frequency ACMF treatment in the first group (with MF) increased release depending on the intensity of the MF. Since drugs with a higher molecular weight should generally be expected to be released slower, DOX (543 gr/mol) demonstrated moderate release kinetics. The next rapid increase in DOX release was observed by re-applying the same MF for 2 hours after three hours intervals (Fig. 6f). The controlled release mechanism could be adjusted by combining plasma treatment with ACMF treatment. This is because plasma deposition of AAc could delay the release process by decreasing release kinetics. In contrast, ACMF treatment greatly increased release kinetics.

### Cancer cells viability and adhesion induced by DOX-loaded MENs

**Figure 7:**
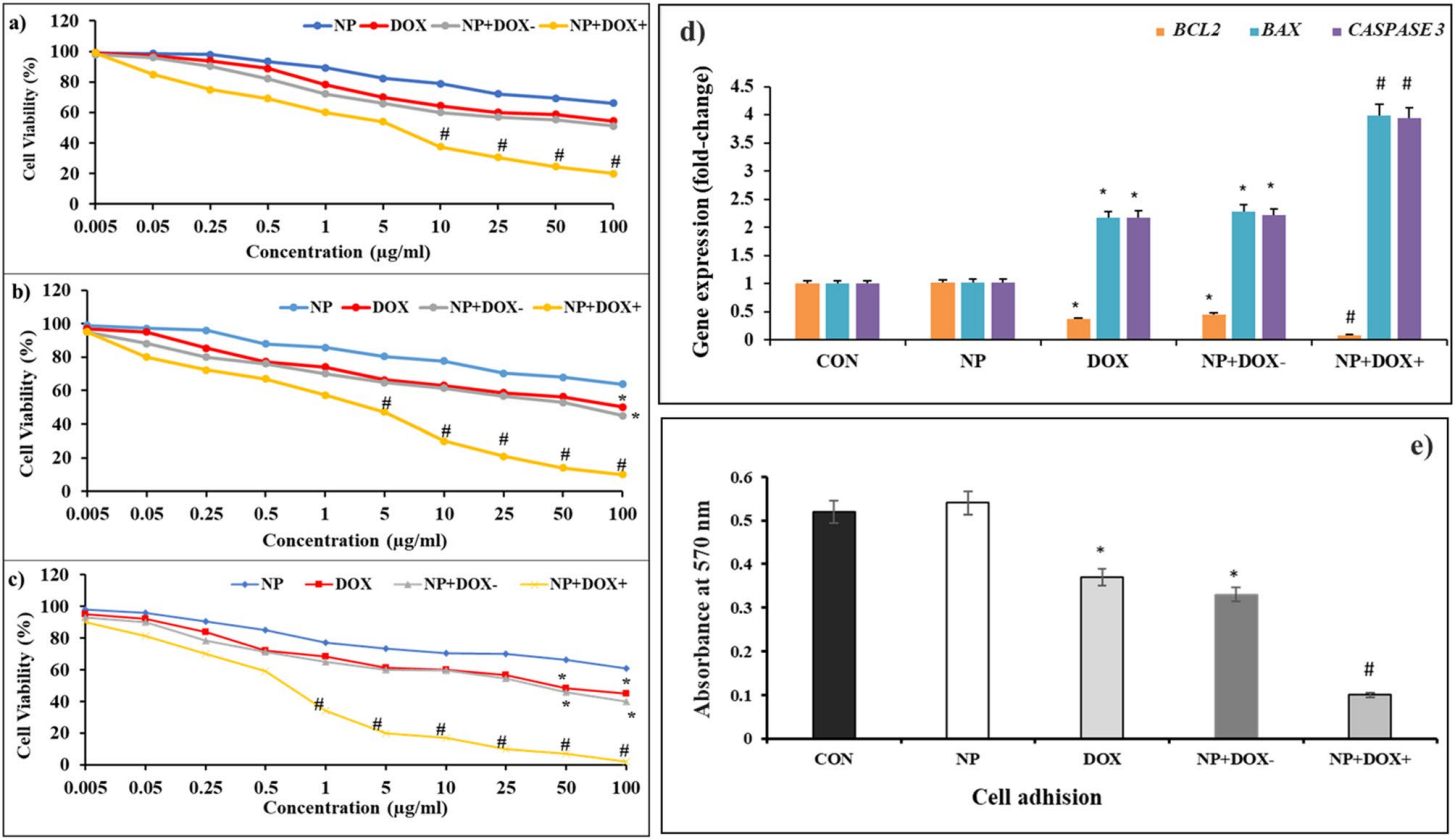
In vitro cytotoxicity of MENs-DOX in 4T1 breast cancer cells. (**a**–**c**) dose-response results of 4T1 cells upon treatment with nanoformulations after 24 h (**a**), 48 h (**b**), and 72 h (**c**) at several concentrations (0.005, 0.05, 0.25, 0.5, 1, 5, 10, 25, 50, 100 µg/mL); (**d**) gene expression levels of proteins associated with apoptosis (*BCL2*, *BAX*, *CASPASE 3*); (**e**) crystal violet absorption quantification of attached cells at 570 nm for MENs, DOX, MENs+DOX−, and MENs+DOX+ with concentrations (20 µg/mL and 30 min). Relative results are normalized to the expression of the control group. (*) indicate significant levels compared to DOX, and (#) indicates a significant level compared to MENs+DOX−. Magneto-electric nanoparticles (MENs = NP).

The viability of 4T1 cancer cells incubated with nanoformulations at 24 h, 48 h, and 72 h was evaluated using MTT assay and the results are presented in Fig. 7a–c. The results show that after 72 h, more than 80% of the cells were still alive. Increasing the concentration of MENs did not reveal a significant effect on viability after 24 h, 48 h, and 72 h incubations. Therefore, MENs did not cause cytotoxicity, even at relatively high concentrations, suggesting they are non-toxic. However, the viability of 4T1 cells treated with MENs+DOX+ drastically decreased compared with MENs+DOX− upon increasing the concentration and incubation time. This can be observed at concentrations ≥10 µg/mL after 24 h, at concentrations ≥5 µg/mL after 48 h, and at concentrations ≥1 µg/mL after 48 h. It was also observed that cells treated with MENs+DOX − had a significant decrease in viability at 100 µg/mL after 24 h and 48 h and 50 µg/mL after 72 h, which is comparable to the DOX-only treated group. It was found that MENs+DOX and ACMF exposures at varying concentrations resulted in a significant synergistic effect on the inhibition of cell proliferation. Combining low-frequency ACMF with DOX-loaded MENs at higher concentrations increases drug uptake, which induces electroporation on the cancer cell's surface. Additional exposure to MF facilitates the breakdown of covalent bonds between drugs and nanoparticles, releasing DOX inside cancer cells that promote apoptosis. Considering that polarized cells have a high potential for cellular adhesion, we next examined the effects of DOX-loaded magnetoelectric nanoparticles (NP) on cell adhesion. Optical absorbance at 570 nm was measured to determine cell adhesion after different nanoformulation treatments (Fig. 7e). The assay was performed to determine the influence of MEN's treatment on the cell adhesion of 4T1 cancer cells. The absorbance of the MENs+DOX+ treated group was significantly reduced, and the combined treatment of ACMF and MENs severely impaired the invasion ability of 4T1 cells.

### Gene expression

Figure 7d shows the expression of caspase3 protein as one of the latest markers of the apoptosis pathway. Additionally, BAX and BCL-2, two cytoplasmic proteins, were investigated, which act as promoters and inhibitors of apoptosis, respectively. Compared to MENs+DOX without MF, MENs+DOX with 45 min MF significantly decreased the expression level of BCL2 and enhanced the expression level of BAX and Caspase 3 genes in 4T1 breast cancer cells. MENs+DOX+ increased caspase 3 levels in a time- and dose-dependent manner. An increased upregulation of caspase 3 was observed upon MENs+DOX+ exposure, which is 4 fold higher than the control and MENs’ groups. Moreover, free-form DOX exhibited 2.5 times higher upregulation in Caspase 3 expression than the control group at 20 µg/mL dose. It has been shown that DOX, MENs+DOX-, and MENs+DOX+ treatment were associated with a decrease in anti-apoptotic BCL-2 mRNA levels and an increase in pro-apoptotic BAX mRNA levels. Across all doses and incubation times, nanoformulations significantly increased BAX/BCL-2 ratios (p < 0.05). The ratio of BAX/BCL-2 reached its highest level (more than 4-fold change) after 48 h incubation with MENs+DOX+.

### Animal study

**Figure 8:**
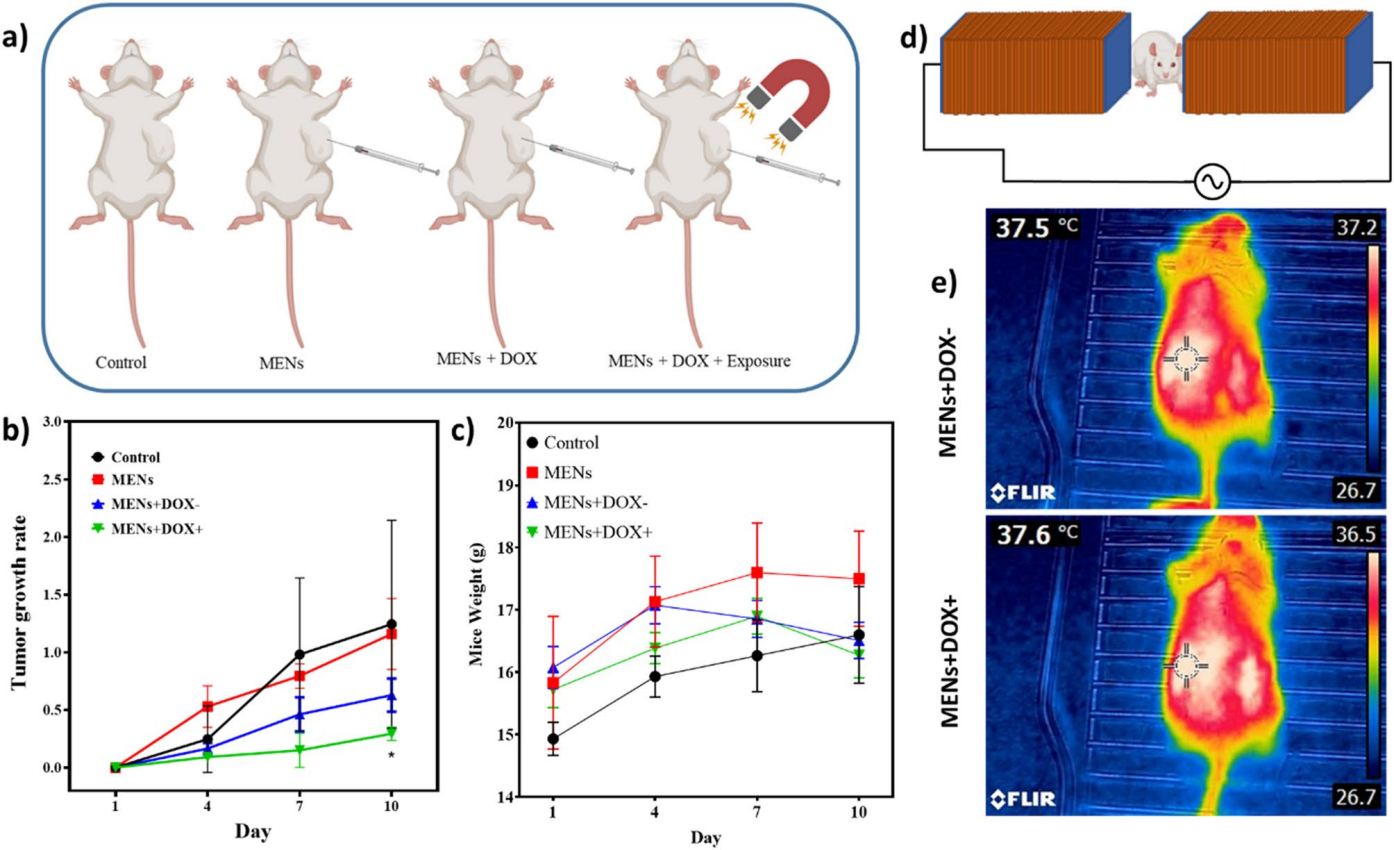
Investigation of MFO-BTO-DOX nanoformulation on 4T1 tumors in vivo. (**a**) scheme of study groups (control, MENs, MENs+DOX−, and MENs+DOX+ for 45 min); (**b**) tumor growth rates; (**c**) mice weight, (*) indicate significant differences compared to the control; (**e**) FLIR thermographs of MENs-DOX-injected mice before and immediately after MF exposure indicate no temperature increase due to low-frequency, low-intensity MF treatment (**d**) confirming absence of hyperthermia.

A schematic illustration of the different treatment groups can be seen in Fig. 8a in more detail. It was observed that the behavior of mice did not change during the last seven days before treatment. All the mice were alive when the experiment started. In our experiments, we injected a stable suspension of MENs and MENs+DOX intratumorally every three days at 100 mg kg^−1^ (about one-third of the tolerable dose). The tumor volume and the weight of the mice were monitored for the next 10 days. The MENs+DOX+ treated group was irradiated for 45 min with an external ACMF by placing the mice inside the parallel electromagnets, as illustrated in Fig. 8d. According to the FLIR thermographic images, there was no significant increase in the tumor site's temperature due to MF treatment in mice before and after MF treatment (Fig. 8e). Indeed, low-frequency, low-intensity MF treatment is insufficient to lead to hyperthermia. The tumor growth rates of all the different groups are shown in Fig. 8b. On the 10th day following treatment, significant tumor growth inhibition was observed in the MENs+DOX+ group (P-value < 0.05). However, tumor growth increased by 125% and 116% in the control and MEN’s groups, respectively, after 10 days of treatment. In contrast, in the treatment group that received target chemotherapy with MENs+DOX+, the tumor volume showed a slow increase of 30% over the same timeframe. In the MENs+DOX− group, tumor growth rates were similar to those of the MENs+DOX+ group but with a faster rate of progression (63% growth rate) due to the slow and continuous release of DOX from MENs. Furthermore, by the 10th day after treatment, the mean tumor weight of the MENs+DOX+ treated group had decreased by about four-fold compared to the control group. These data show the feasibility and effectiveness of nanoformulation for treating breast tumors. As shown in Fig. 8c, the mice did not lose significant weight, indicating that the MENs used in our study were safe to administer.

## Discussion

In this study, we synthesized DOX-loaded MnFe_2_O_4_-BaTiO_3_ NPs by *in situ* co-precipitation of MnCl_2_ and FeCl_3_ in distilled water in the presence of sodium hydroxide followed by BaTiO_3_ coating to increase the biocompatibility, stability and drug loading capacity of the synthesized sample. Following that, DOX as a targeting moiety was conjugated to the carboxyl, carbonyl, or hydroxyl functional groups on the surface of MENs through plasma polymerization of acrylic acid.

It was confirmed by FTIR, XRD, TEM, and Raman spectroscopy that perovskite-coated manganese ferrite nanoparticles were synthesized. Their physical characteristics were similar to those described in the literature. The average size of our MENs was 80 nm, with a size distribution in the range of 60–110 nm. An ideal particle size for NPs to improve blood circulation was 10–100 nm^35^. Considering these factors, the sample prepared in the current study has the right dimensions for biomedical applications. The VSM analysis indicates that the Ms of MnFe_2_O_4_ spinel is 38 emu·g^−1^. In addition, the room temperature magnetization curves of the spinel core in our experiment exhibit very low hysteresis, demonstrating its superparamagnetic behavior. As a result of the superparamagnetic property, the particles become magnetized only when exposed to an external magnet, which is highly valuable. By attaching an MF, they can be easily separated from their media. Additionally, without an MF, they exhibit no magnetization and can disperse efficiently in a mixture. This allows fast diffusion of substrates onto the nanoparticle surface active sites. In our VSM results, Ms decreases when a shell and drug are added. It has been reported that, as MEN's size increases, the coercivity (Hc) increases to a maximum, then declines towards zero when the state of ferromagnetism in the single domain reaches a maximum^36^.

The zeta potential of non-treated MENs is obtained around − 2.5 mV, while its value decreases to − 20 mV and − 37 mV by applying 100 and 200 Oe ACMF treatment, respectively. With an external MF, MENs could generate relatively strong dipole electric fields locally and decrease their microenvironment resistance. Therefore, they show a reduced real impedance under field exposure. Our EIS findings indicate the presence of two depressed semicircles, each linked to distinct relaxation processes in the high- and low-frequency ranges, as corroborated by Ref.^37^. The high-frequency semicircle is attributed to the bulk properties of the nano-composite, while the low-frequency semicircle arises from the interfaces. Our MEN's impedance spectra can be modeled using two constant phase elements (CPE) parallel to a series-connected resistance, accurately representing the high- and low-frequency relaxation distributions. VSM results also indicated that a large change in the magnetization of the ferrite would occur at a field of approximately 7.5 kOe. This change would be most apparent at this MF. Higher MF results in very small changes in magnetization, while smaller fields result in significantly increased impedance responses at all temperatures^38^.

In physiological media, unmodified nanoparticles tend to aggregate and lack stability. It is necessary to apply a suitable coating to their surface to improve their stability and prevent aggregation under such conditions. Due to its highly reactive ion bombardment, a short-term (45 s) Ar plasma has been used for surface modification of MENs within the etching process. In this process, high-energy Ar ions impinge on the substrate surface, which is placed on the plasma sheath, causing a change in surface roughness and increasing physical adsorption. To modify surfaces, very low-temperature plasma for short-term treatment is preferred, while higher-intensity plasmas are used to sterilize and inactivate surfaces^39^.

Further surface modifications were achieved by using AAc mixture polymerization. In situ OES analysis revealed that plasma polymerization proceeds primarily via opening the C=C bonds in the AAc molecule structure. As a result, the MEN's surfaces exhibit active species that strongly retain the carboxylic acid groups. Notably, breaking the C=C bond only requires 2.74 eV, while breaking the C–C, C–O, and C=O bonds necessitates 3.61, 3.64, and 7.55 eV, respectively^40^. Therefore, it is more feasible to break the C=C bond easier. This produces –COOH, –OH, or –C=O groups. Then it can be predicted that plasma polymerization of CH_2_=CHCOOH first dissociates to C=O, O–C=O, C–O–C, –HC=O fundamentals according to the following possible reactions:

H_2_C=CHCOOH → H_2_C=CH+COOH

H_2_C=CHCOOH → H_2_C=CHCO+OH

H_2_C=CHCOOH → H_2_C=CH_2_+CO_2_

H_2_C=CHCOOH → H_2_C=CHOH+CO

Particularly when CH_2_ = CHCOOH is admixed with O_2_, the flow rate ratio plays a key role in avoiding unwanted products. By increasing the O_2_/AAc ratio higher than 2, there is a high probability of producing CO_2_ and H_2_O based on the following reaction:

C_3_H_4_O_2_ + 3O_2_ → 3CO_2_ + 2H_2_O

FTIR spectra in Fig. 4d reveal the presence of these products in our results. DOX contains an amino group (-NH_2_) on the six-membered ring, which can conjugate with carboxyl (-COOH), and carbonyl (-C=O) groups on another six-membered ring^29^. These are the two most common conjugating sites for prodrug design^41^. As a result, the -OH and -COOH groups on the functionalized MENs can form a strong hydrogen-bonding interaction with the -OH and -NH_2_ groups in DOX. Also, due to the pre-activation of Ar, DOX can adhere noncovalently to the MENs' surface because of its physical nanoscale roughness and electrical negative charge. In our experiments with low initial DOX concentrations, we obtained 71% loading capacity by Ar pretreatment followed by AAc+O_2_ polymerization in a pulsed mode.

A part of the uptake DOX were quickly released by ACMF (Fig. 6f) indicating a weaker bond due to the physical adoration, however the low-intensity, low-frequency field enhanced penetration into cancer cells by increasing nano electroporation. However, a controlled release mechanism could be adjusted by combining plasma treatment with ACMF treatment. This is because plasma deposition of AAc could delay the release process by decreasing release kinetics. In addition, ACMF treatment greatly increases release kinetics. In this case, the drug release of MENs is controlled by a passive mechanism followed by external MF treatment. This has been described intelligibly in other documents^20, 42–44^.

The MTT assay was performed to investigate the cytotoxicity of the nanoparticles on 4T1 cancer cells, and the results indicated that a time-dependent reduction in viability of the cells treated with MENs+DOX+ was observed. The dose-dependent inhibitory effects of MENs+DOX+ on the cells were also observed, and low doses of MENs efficiently attenuated cell viability. Our MENs did not induce inadvertent hyperthermia *in vitro* or *in vivo* since low-intensity, low-frequency MF does not produce significant heat. Additionally, MENs' mechanical vibration exposed to MF is not sufficiently frequent to cause localized heat to develop in tumor regions. A temperature rise is usually associated with MF of 20 mT or higher^45–47^. It was therefore hypothesized that heat-induced cellular death in vitro was not caused by 10 mT (100 Oe) MF, which is also confirmed in our study by the FLIR picture depicted in Fig. 8c, with and without MF treatment for 45 min. Additionally, MF did not affect the viability of non-treated cells. It is reported that at a very high intensity (200–2000 mT) for an extended period, MF can cause DNA damage to cells, enhance caspase expression and manipulate metabolic activity^48^. The present study used low-intensity external MF (10 mT) for targeted drug delivery for a short time. Therefore, the control groups in our study did not show cytotoxicity due to MF.

We also found an increase in cell adhesion after MENs+DOX+. According to Shahzad's findings^49^, the morphology of HepG2 cells changes when exposed to free drugs and drug-loaded MENs. The cells exhibit elongation and fibrous characteristics, which could affect their metastatic potential by altering cellular adhesion and invasion processes. Betal reported that, under ACMF excitation (50 Oe, 60 Hz), the MENs act as a localized electric periodic pulse generator^10^. This MF intensity is sufficient to vibrate 100 nm MENs 2–3 orders of magnitude smaller than cancer cells.

**Figure 9:**
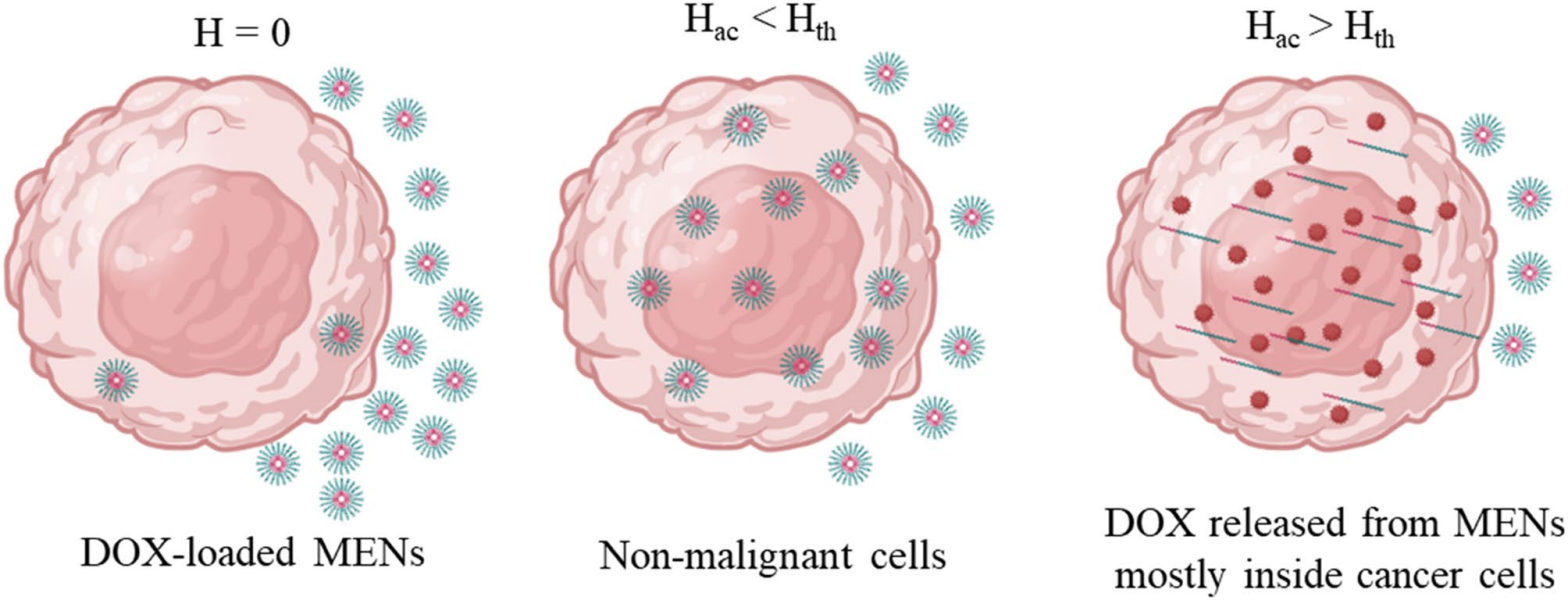
Schematic illustration of hypothesized anti-cancer drug release using MENs in the vicinity of cancer cells. This physical phenomenon is caused by electric field interactions (i) between MENs and drugs and (ii) between drug-loaded MENs and cells. The electric properties of the membrane enable MENs to differentiate between cancer cells and normal cells, as cancer cells exhibit a markedly lower threshold field for electroporation. By utilizing the high-specificity nano-electroporation effect, DOX-loaded MENs can infiltrate tumor cells when they come in proximity to the cell membrane surface. In contrast, normal cells remain intact due to a significantly higher nano-electroporation threshold field.

Developing anti-cancer drugs that release specifically on tumor cells while sparing normal cells is a major challenge. By controlling the electric field surrounding tumor cells, magnetoelectric nanoparticles have proposed a solution^48^. Due to ions (such as K^+^, Na^+^, and Cl^-^), the cell membrane is regarded as an electrically polarizable medium. Concentrations of these typical ions change gradationally between the intracellular and extracellular environment. Consequently, an electrical field can change membrane porosity, a process known as electroporation (Fig. 9). External MF treatment on drug-loaded MENs can generate sufficient electric fields near the cancer cell membrane, allowing them to penetrate and deliver drugs^44^. Interestingly, MENs can differentiate between normal and cancerous cells. This cancer cell death selectivity is because of the threshold field required for electroporation on normal and cancerous cells (about 3 mT and higher than 20 mT, respectively), which demonstrates a physical mechanism behind their specificity towards cancer cells^21, 44, 50^

.

## Materials and methods

### MENs' preparation

According to reported standard protocol, MnFe_2_O_4_ spinel as the nano-carrier core was prepared following the co-precipitation of MnCl_2_ and FeCl_3_ in distilled water in the presence of sodium hydroxide^51^. Briefly, MnCl_2_.4H_2_O and FeCl_3_.6H_2_O were taken in the molar ratio of Mn^2+^: Fe^3+^ = 1:2 to obtain 0.3 mol.L^-1^ metal ion solution of 100 mL containing 0.1 mol.L^−1^ Mn^2+^ and 0.2 mol.L^−1^ Fe^3+^. This solution was slowly dropped into 100 mL 3 mol.L^−1^ of NaOH solution at the preheated temperature of 95 °C and was continuously stirred for 2 h. The mixture was then filtered, washed, and dried for 12 hours at 60 °C. According to Rodzinki's report^21^, 0.03 g of BaCO_3_ and 0.1 g of citric acid were first dissolved in 30 mL of distilled water. This is done to prepare the BaTiO_3_ perovskite forerunner solution. Then, 0.05 mL of titanium (IV) isopropoxide (TTIP) and 1 g of citric acid were dissolved in 30 mL of ethanol. Both solutions were added to 0.1 g of dry MnFe_2_O_4_ core nanoparticles and sonicated for 2 h to fully disperse the cores in the shell forerunner solution. The solution evaporated with continuous stirring at 200 rpm and 70 °C overnight. The resulting mixture was sonicated at 60 °C for 1 h. The gel-like mixture was transferred to a heat-resistant ceramic dish, transferred to a furnace at 800 °C for 5 hours, and cooled overnight at a constant rate for the calcination process. After 15 min of grinding, the MnFe_2_O_4_-BaTiO_3_ core-shell was formed.

### Alternating current magnetic fields (ACMF)

A homogeneous MF was generated in the interspace between 2 coils (n=1000, L=0.046 H, R=0.6 Ω) filled with iron cores. The coils were connected to a power supply (Chroma, 61604), and the samples were placed in the gap. An approximately homogeneous MF with a 2.5 × 2.5 × 1 cm area was obtained by applying V = 50V, I = 3–4 A, f = 0 Hz for DC mode, and f = 100 and 200 Hz for low-frequency AC mode. The average MF strength is obtained using a Tesla meter (Magfine).

### MENs' characterization

The synthesis of the magnetic core, perovskite shell, magneto-electric nanocarriers, and functional groups on their surface was investigated by Fourier Transform Infrared (FTIR) spectroscopy. The samples were prepared in KBr medium (1:20), and spectra were recorded in the range of 400–4000 cm^−1^ using an FTIR spectrometer (TENSOR 27, Bruker, Germany). The phase formation and crystallographic state of spinel, as well as MENs and MENs with the drug, were determined with an STOE-IPDS II single crystal X-ray diffractometer (XRD) with Ni-filtered Cu-Ka radiation (l = 1.54 A˚). Magnetic measurements were performed utilizing a vibrating sampling magnetometer (VSM) instrument (VSM, LBKFB model-Meghnatis Daghigh Kavir Company, Iran). The MENs' size diameter and distribution were determined using the dynamic light scattering (DLS) technique by Zetasizer (Nano ZSP, Malvern Panalytical). The nanoparticles were thoroughly dispersed in an ethanol bath via ultrasonication for 15 minutes (0.5 mg/mL). The samples were measured in triplicate at 25 °C for identification. Additionally, Raman spectroscopy (Teksan, Iran) was conducted to confirm the synthesis of nanoparticles and characterize them. A 50 μl droplet was dropped on a silicon wafer that was washed 3 times for 10 min in an ultrasonic bath with alcohol, ethanol, and distilled water, respectively, and then dried in a nitrogen atmosphere. The size and morphology of the nanoparticles were observed by field emission scanning electron microscopy (FE-SEM) (Hitachi SU3500) and transmission electron microscopy (TEM) (JEOL JEM-2100 Plus 200kV, LaB6 filament). After drying, a 50 μl droplet was dropped on a clean glass slide and coated with sputtered gold. Band gap energy of MENs was obtained from the interpolation of UV-vis absorption spectrum of 1mL prepared solution in ethanol at 200–1100 nm (AvaSpec 3648 fiber optic spectrometer equipped with deuterium lamp).

### Magnetoelectric properties of MENs

The Zeta potential of MENs, MENs pre-activated with Ar plasma, and MENs treated with AAc polymerization with and without ACMF exposure is measured by DLS (Nano ZSP, Malvern Panalytical). 0.5 mg/mL of each sample was sonicated for 15 minutes and analyzed immediately to avoid agglomeration. An MF of 20 mT and 200 Hz was then applied to the samples for 10 minutes. Electrical impedance spectroscopy (EIS) is another useful technique employed to investigate the electrical behavior of magnetoelectric nanomaterials under MF exposure^37^. A vector network analyzer (Bode 100, OMICRON LAB) was employed to evaluate the electrical behavior of MENs under AC field exposure (Fig. 3a). The device was connected to a homemade chip of two thin planar electrodes on an electronic board to inject the samples. This chip was connected to a vessel with a height of 10 mm and a diameter of 8 mm that served as a chamber to receive the samples. Next, MENs in ethanol at 5 mg/mL were injected into the chip. An AC electric signal was applied to the electrodes, and the ACMF was simultaneously applied to the sample through the electromagnets. The MENs' Nyquist spectrum was measured at frequencies from 1 kHz to 50 MHz. The magnetoelectric behavior of MENs can be understood from the changes in Cole-Cole diagrams under ACMF exposure. The experiments were carried out at room temperature.

#### Low-pressure radio frequency plasma setup

A low-pressure capacitively coupled (CCP) radio frequency (RF) plasma was employed for liquid-free plasma surface modification of MENs (Fig. 4a). A cylindrical stainless-steel chamber 40 cm in diameter and 35 cm in height equipped with a lateral visible window for optical analysis was used in our structure. The powered copper electrode is connected to the RF power supply (13.56 MHz and 0–400 W) through a matching network, and the chamber's body serves as the ground electrode. Two rotary and ruts vacuum pumps connected in series were working to evacuate the air up to ~5mTorr. Mass flow controllers (MFC) (ALICAT) fed different gases to the chamber. Each experiment had 0.5 mg of MENs placed on the powered electrode in a clean glass petri dish. Additionally, the process pressure was monitored during the experiments using a Pirani gauge.

#### Pre-activation using Ar plasma

Pre-activation of MENs was carried out using Ar plasma treatment. A 20 sccm Ar (99.99%) was fed to the chamber at a pressure of 50 mbar (Table 1), and then the MENs were activated with 75 W RF power in a continuous wave (CW) mode. To optimize the pre-treatment exposure time's effect on physical surface roughness, a 50 nm thin film of BaTiO_3_ deposited on quartz glass was used as a model of a perovskite shell and treated by the same Ar plasma applied for MENs. Samples were treated at 30, 60, 180, and 300s 3 times, and their morphology and average roughness (S_q_) was measured by atomic force microscopy analysis (AFM) (nano Surf).

#### MENs' functionalization by AAc plasma polymerization

Acrylic acid (C_3_H_4_O_2_) (Merck) was used as a monomer precursor for the plasma-enhanced chemical vapor deposition (PECVD) process to produce carboxyl and carbonyl-rich surfaces. 1 mL AAc was injected into the bubbler, and its molecules were transferred to the plasma zone through Ar carrier atoms (Fig. 4a). Air, oxygen (99.995%), AAc, and the mixture of AAc and O_2_ with different ratio plasmas were investigated to determine their ability to produce hydroxyl (-OH), carbonyl (–C=O), and carboxylate (–COOH) functional groups. All samples were pre-activated with Ar plasma (75 W and 50 mTorr) for 45 s, considered the optimum treatment time. The operating conditions for these experiments are listed in Table 1. AAc polymerization experiments were performed in pulsed mode with a pulse duration of 100 ms (50% duty cycle). The composition of the gas phase plasmas, including reactive species, radicals, and ions, was analyzed by real-time optical emission spectroscopy (AvaSpec3648-USB2) in the 200–1100 nm range. Additionally, FTIR spectra of the MENs-treated plasma (Table 1) were obtained to confirm the gas phase generated functional groups on the surfaces.

### Drug loading

In order to identify doxorubicin's optical properties and to determine a calibration curve, ultraviolet-visible and fluorescent spectra were performed at various concentrations of Doxorubicin (Merck, 2 mg/mL) (DOX—MW 543). To prepare DOX-loaded MENs, 2 mg of plasma-treated nanoparticles were dispersed in 800 μl PBS and sonicated for 15 min. Then, 200 μl DOX (2mg/mL) was added to the solution and incubated for 24 h at 200 rpm and 37 °C in dark shield condition. The solution was then centrifuged at 9000 *rpm* for 5 min, and the MENs were collected and separated from the supernatant magnetically. The generated MENs were washed 3 times with distilled water to remove any unbound DOX^52^. FTIR spectroscopy was carried out to confirm the conjugation of DOX to the MENs, and the thermogravimetric assay (TGA) reports the percent weight loss by temperature. The MENs were heated up to 300 °C (10 °C/min), and their weight loss fraction was recorded using TGA-DTA and plotted as a function of temperature. Furthermore, the concentration of unbonded DOX in the supernatant was measured by UV-Vis spectrometry at λ_excitation_ = 480 nm and λ_emission_ = 590 nm. The drug loading efficiency of MENs was calculated by Eq. (1):

1 $$\mathrm{Drug\; loading\, \%}= \left(\frac{\mathrm{Initial\; drug \;concentration}-\mathrm{Drug \;concentration\; in\; supernatant \;solution})}{\mathrm{Initial \;drug \;concentration}} \right)\times 100.$$

### Drug release

The drug-loaded MENs were dispersed in 1 mL PBS and transferred into an 18 kDa dialysis bag to assess the release of Dox into the phosphate-buffered saline solution (PBS, pH 7.4). The dialysis bag was enclosed and kept in a 50 mL falcon with PBS solution. Following the placement of samples on the shaker, one group of samples was exposed to MF twice for 7 hours, each time for 2 hours. Each falcon was taken up and replaced with 1 mL of fresh solution. Finally, samples were centrifuged at 5000 *rpm* for 5 min and analyzed using spectrophotometry (λ_excitation_ = 480 nm and λ_emission_ = 590 nm). The percentage of drug released in the samples was estimated using Eq. (2), and the plot of the cumulative drug against time intervals presents the release kinetics of drugs from each synthesis nano-formulation in the presence and absence of applied ACMF.

2 $$Drug\, release\, \%=\left(\frac{Absorbance\, of \,supernatent}{Absorbance\, of\, drug \,used}\right)\times 100.$$

### In vitro studies

4T1 murine breast cancer cells were cultured in NG (4.5 g/L glucose) RPMI-1640 medium supplemented with 10% fetal bovine serum (FBS) (Gibco Co, USA), 2% L-Glutamine, 100 U/mL penicillin, and streptomycin solution (Sigma- Aldrich, USA) that was maintained at 37 °C, 5% CO_2_ and 95% humidity. The cells' growth in the medium was monitored using an inverted microscope. The 5×10^4^ 4T1 cells were seeded in a 96-well plate and were incubated for 18 hours. Each nanoformulation was then applied to cells at concentrations of 0.005, 0.05, 0.25, 0.5, 1, 5, 10, 25, 50, and 100 μg/mL for 24, 48, and 72 h. The cells were divided into five groups, including (1) untreated cells (control), (2) cells treated with DOX, (3) cells treated with MENs, (4) cells treated with DOX-loaded MENs without ACMF exposure (NPs+DOX-), and (5) cells treated with DOX-loaded MENs with ACMF exposure (NPs+DOX+). For a better comparison, the concentration of free DOX in group 2 was equal to the concentration of drugs bound to nanoparticles in the groups 4 and 5. Also, the cells of group 5 were treated for 30 min with ACMF treatment at 100 Oe and 100 Hz. The cells’ supernatant was removed, each plate was filled with 200 µl medium containing 0.5 mg/mL MTT solution, and the cells were incubated for 4 hours. The fluorescence was measured using a microplate reader (Tecan, Germany) at 570 nm, and viability was calculated using Eq. (3):

3 $$\mathrm{living\, cells}\left(\mathrm{\%}\right)=\left(\frac{{\mathrm{A}}_{\mathrm{sample}}}{{\mathrm{A}}_{\mathrm{control}}}\right)\times 100.$$

A_sample_ and A_control_ are the absorbances of treated and control cells at 570 nm. Following 24 h treatment with optimal concentrations of nanoformulations, the cells were rinsed with PBS, then fixed using 100% ethanol for 10 min. Thereafter, cells were stained with crystal violet (25% (v/v) in 0.1% methanol (w/v)), washed with distilled water 5 times, and dried at room temperature. Finally, the cells were leased with a 0.2% triton X-100 solution, and their absorbance was measured at 570 nm to evaluate cell adhesion strength after different treatments^53^.

### Real-time PCR

Total RNA samples were first extracted by triazole according to the manufacturer’s instructions. The cDNA was then synthesized in a total volume of 20 μL with a commercial kit (Fermentas, Lithuania) at 40 °C for 60 min corresponding to the manufacturer’s instructions. Real-Time PCR (TaqMan) was used for the quantification of relative gene expression according to the QuantiTect SYBR Green RT-PCR kit from Takara Bio Inc. (Japan). β-actin expression was used to normalize genes as the Internal Reference Gene. Table 2 shows the list of primers. The qRT-PCR reactions were performed with an ABI StepOne Plus System (Applied Biosystems; Thermo Fisher Scientific, Inc.). The expression level of the genes was calculated according to the 2−ΔΔCT method^54^.

**Table 2:** The primer sequences utilized for gene expression analysis.

Genes	Forward	Reverse	Tm	Length
*BAX*	5´- CCGGCGAATTGGAGATGAACT -3´	5´- CCAGCCCATGATGGTTCTGAT -3´	60	229
*BCL2*	5´- GAGCCTGTGAGAGACGTGG -3´	5´- CGAGTCTGTGTATAGCAATCCCA -3´	59	196
*CASPASE 3*	5´- CTCGCTCTGGTACGGATGTG -3´	5´- TCCCATAAATGACCCCTTCATCA -3´	59	201
*GAPDH*	5´- AATGGATTTGGACGCATTGGT -3´	5´- TTTGCACTGGTACGTGTTGAT -3´	58	213

### Animal study

6–8 weeks old, female inbred Balb/C mice (weighing 15–18 g) were purchased from the Pasteur Institute of Iran. Mice had free access to food and water and were kept in a 12-light/dark cycle. The 1.5×10^5^ 4T1 cells were suspended in PBS (100 μl) and then subcutaneously co-transplanted into the flank of the animals. After 10 days, tumors appear, and treatments begin when the tumor volume reaches 150–200 mm^3^ (day 12). Mice were divided into 4 groups (n=6) randomly, including (1) control without any perturbation, (2) MENs without DOX, (3) DOX-loaded MENs without external MF exposure, and (4) DOX-loaded MENs and exposure to ACMF. The study lasted 24 days, beginning at the time of administration and ending when the animals were euthanized in groups 2, 3, and 4 and were treated every 3 days for 12 successive days (Fig. 8a). Each mouse was treated at 2 mg/kg in 50 μl PBS intratumorally, and group 4 was exposed for 45 min to ACMF (2oo Oe, 100 Hz). Mice's tumor size and body weight were measured every three days. To calculate tumor volume by digital caliper, the maximum longitudinal diameter, the greatest transverse diameter, and the depth from the body to the highest point of the tumor were measured. The modified ellipsoidal formula (Eq. (4)) was used for tumor volume calculation. The animals were immediately euthanized via cervical dislocation at the end of the study.

4 $$\mathrm{Tumor \,volume}=\frac{\pi }{6}\times (\mathrm{length }\times \mathrm{ width}\times \mathrm{depth}).$$

### Statistical analysis

Statistical analyses were performed for each experiment, and appropriate tests were chosen based on the type of data and research question. Data normalization was performed using Excel 2016 (Microsoft, Redmond, WA, USA). For physical characterizations, the data was analyzed and expressed using Origin 2020 software (OriginLab Corporation, Northampton, MA, USA) as mean ± standard deviation to evaluate the relationship between variables. Data analysis and graphing were conducted for in vitro and in vivo experiments utilizing Excel and Prism 8 (GraphPad Software, San Diego, CA, USA). One-way analysis of variance (ANOVA) or t-test was performed to determine the degree of statistical significance between groups. Significance levels were set at a p-value < 0.05 for all analyses.

### Ethical approval

All methods were performed under the relevant guidelines and regulations. All procedures performed in animal studies were conducted within the international guidelines of the Weather all report and the national guidelines of the Institutional Animal Care and Use Committee (IACUC). The Ethics Committee of Tehran University of Medical Sciences has approved the project (NO: IR.TUMS.VCR.REC.1397.082). Reports concerning experimental animals follow the recommendations in the ARRIVE guidelines. The authors confirm that there are no human samples/cells involved in the study.

## Conclusion and outlook

In this study, we report a straightforward and effective approach for synthesizing MnFe_2_O_4_@BaTiO_3_ magnetic nanoparticles that are functionalized through low-pressure AAc plasma polymerization for drug delivery applications. The functionalized nanoparticles with an average diameter of 80 nm exhibit superparamagnetic properties, with a saturation magnetization of 20 emu·g^−1^. Through our experiments, we achieved a loading capacity of 71% by Ar pretreatment, followed by AAc+O_2_ polymerization within a pulsed mode RF discharge. The AAc+O_2_ plasma has shown significant potential for generating COOH and C=O functional groups through a polymerization process. Our findings suggest that the oscillating magnetic field triggers increased cytotoxicity via electroporation mechanisms. Low-pressure plasma processing has proven to be a fast, repeatable, chemical-free, but relatively expensive method for modifying biomaterial surfaces. We suggest using atmospheric pressure PECVDs based on dielectric barrier discharges (DBDs) to address this. Moreover, mechanical vibration or external magnetic vibrators can enhance functionalization by facilitating plasma-NP interaction in a more homogenous manner. Additionally, internal DC pulses (bias) on the powered electrode efficiently improve the overall retention time of NPs in the plasma zone. Carboxylic activation using a coupling agent such as N-hydroxysuccinimide (NHS) and N-(3-dimethyl aminopropyl)-N’-ethyl carbodiimide hydrochloride (EDC) after plasma polymerization is suggested to achieve more efficient conjugation of the drug to the nanoparticles. Lastly, further preclinical evaluations and human trials will be required to reveal the efficacy of plasma-functionalized MENs as anticancer drug delivery systems.

## Data Availability

The data supporting this study's findings are available from the corresponding author, AMA, upon reasonable request.
